# Nonlinear relationship between sleep quality and prevalence of type 2 diabetes: A cross-sectional study of community patients with chronic diseases

**DOI:** 10.1371/journal.pone.0356250

**Published:** 2026-08-21

**Authors:** Juan Ge, Peifang Lu, Shuzhi Peng, Haiyun Huang

**Affiliations:** 1 College of Health Management, Shanghai Jian Qiao University, Shanghai, China; 2 Zhangjiang Community Healthcare Center, Shanghai, China; University of Montenegro-Faculty of Medicine, MONTENEGRO

## Abstract

**Objective:**

To delve into the intricate relationship between sleep quality and prevalence of type 2 diabetes (T2D) among community-dwelling chronic disease patients, and to provide insights that can inform clinical practice.

**Methods:**

Data on basic information and sleep quality of patients with chronic diseases were collected via questionnaire surveys. Following descriptive and logistic regression analyses, restricted cubic spline regression was further applied to explore a possible nonlinear dose-response relationship between sleep quality and prevalence of T2D among these patients.

**Results:**

A total of 902 valid samples were included in this study, among which 565 were cases of T2D, accounting for 62.64%. The average score of the Pittsburgh Sleep Quality Index (PSQI) was 11.11. In all three models, a positive association was observed between higher PSQI scores and prevalence of T2D. Subsequent analysis employing RCS regression substantiates this nonlinear association and discerns two inflection points at 12 and 13 When the PSQI score was greater than 12, the odds ratio (OR) increased with higher PSQI scores, suggesting a significant increase in the association with the prevalence of T2D. When the PSQI score reaches 13, the OR stabilises, indicating that the rate of increase in risk has slowed.

**Conclusion:**

Sleep quality is an important factor for the prevalence of T2D, exhibiting a threshold range effect. Community health practitioners may consider sleep quality assessment as a supplementary tool, particularly in patients with existing chronic conditions. Implementing targeted sleep improvement interventions in this high-risk subgroup (PSQI≥12) may holds substantial potential to be associated with reduced T2D prevalence.

## 1. Introduction

Type 2 diabetes (T2D) is the predominant form of diabetes on a global scale, and its prevalence continues to increase in all countries. The data published by the International Diabetes Federation (IDF) shows that more than 580 million people worldwide live with diabetes, with the number expected to surge to 853 million by 2050 [1]. It is noteworthy that patients with T2D account for over 90% of these cases. China has the highest global burden of diabetes, and rapidly evolving lifestyle with an increasingly aging population continues to drive the rising prevalence of T2D [2]. Diabetes is characterized by persistent elevated blood glucose levels and a range of severe systemic complications, including diabetic nephropathy, retinopathy and cardiovascular disease. These collectively have a detrimental effect on patients’ quality of life and life expectancy [3]. Furthermore, the management of T2D necessitates protracted medical interventions and an ongoing financial commitment, thereby imposing a substantial socioeconomic burden on healthcare systems and affected families [4,5]. Parker et al.[4] revealed that the healthcare expenditures for patients with diagnosed diabetes were 2.6 times higher than the anticipated expenses for non-diabetic patients. These significant implications underscore the necessity for in-depth research on the pathogenesis and modifiable risk factors of T2D, which are imperative for the development of effective prevention strategies and the improvement of disease management programs.

A mounting body of evidence indicates a significant clinical association between sleep quality and the development of T2D [6,7]. Sleep is an important physiological process that plays a key role in maintaining normal metabolic function and endocrine homeostasis [8]. Contemporary research suggests that sleep-related disturbances, including short sleep duration, impaired sleep architecture and circadian rhythm dysregulation, may increase the risk of T2D through a variety of pathophysiological mechanisms [7]. The mechanisms implicated in this process encompass reduced insulin sensitivity, dysregulated glucose metabolism, and chronic low-grade inflammation [9].

Research indicated that individuals who sleep for duration less than 7 hours or more than 8 hours per night are predisposed to a higher risk of developing T2D. Furthermore, it has been demonstrated that poor sleep quality and chronic nighttime patterns are significantly associated with increased susceptibility to T2D. It is noteworthy that daytime naps lasting more than 30 minutes have been associated with a 7% to 20% increased risk of developing diabetes compared to naps of shorter duration or none at all [7]. Notwithstanding these advances, there are manifest limitations to the current research paradigm. The majority of studies have used linear regression models founded upon population-level assumptions, a practice which may result in an oversimplification of the complex, non-linear dynamics that exist between sleep parameters and metabolic outcomes. This methodological limitation underscores the necessity for advanced computational methodologies, including machine learning algorithms and spline regression models, to more accurately delineate the dose-response relationship and ascertain critical thresholds for the pathogenesis of sleep-related diabetes.

Individuals with chronic disease constitute a critical demographic for diabetes prevention and management strategies, yet the association between sleep quality and diabetes risk in this population remains inadequately explored [10]. Pathophysiological analyses reveal that the inherent biological predisposition toward metabolic dysregulation in patients with chronic conditions amplifies their susceptibility to diabetes development [11,12]. Wang [13] and colleagues demonstrated that, following adjustment for pertinent variables, hypertensive patients exhibited a 2.956-fold increased risk of diabetes compared with non-hypertensive subjects.Implementing a bidirectional Mendelian randomization analysis, Wang and colleagues [14] established causal evidence that chronic obstructive pulmonary disease (COPD) confers a 6% incremental risk for T2D development (OR 1.06, 95% CI 1.01–1.11; P = 0.006). Concurrently, patients with chronic diseases often experience significant disruption of their sleep architecture due to the dual impact of disease symptoms (such as pain and dyspnea) and therapeutic medications (such as glucocorticoids), thereby falling into a vicious cycle of “disease—sleep disturbance—metabolic deterioration” [15,16]. Clinically, sleep disturbances in these patients are often simplistically attributed to the symptoms of their underlying diseases, which results in the underestimation of the significance of sleep disturbances as an independent risk factor. The existing studies, mostly based on healthy populations, may not accurately reflect the unique characteristics of individuals with chronic diseases. Consequently, there is an urgent need for in-depth studies in chronically ill populations to more accurately assess the impact of sleep quality on diabetes risk in this group.

The restricted cubic spline (RCS) approach provides a flexible regression framework for modeling potential nonlinear associations between continuous predictors and outcome variables, overcoming limitations of linearity assumptions [17,18]. In the context of an investigation into the association between sleep quality and the prevalence of T2D in patients suffering from chronic diseases, the proposed model has the capacity to accurately reveal the complex non-linear relationship between the two, thereby circumventing the potential bias that may be introduced by assuming a linear relationship. The RCS model facilitates a more precise analysis of the impact of sleep quality on T2D risk across a range of values, thereby providing a valuable instrument for gaining insight into the underlying relationship. Furthermore, the model has the capacity to adjust for potential confounders, thereby enhancing the reliability and accuracy of the study results.

Consequently, the primary objectives are to investigate the association between sleep quality and the prevalence of T2D in patients with chronic diseases using the RCS approach. The secondary objective is to determine the critical sleep parameter thresholds that may affect the prevalence of T2D, and to provide a more scientific theoretical basis for clinical intervention and disease prevention.

## 2. Materials and methods

### 2.1. Participants

From 5 January to 30 March 2025, a cohort of patients was recruited from community hospitals in Jiangsu and Shanghai. The inclusion criteria were as follows: (1) age of 18 years or older; (2) presence of chronic diseases such as hypertension, diabetes, cardiovascular diseases, and chronic obstructive pulmonary disease (COPD). The exclusion criteria included: (1) withdrawal during the study period; (2) incomplete key information (e.g., age, gender, height, weight); (3) severe mental disorders; (4) severe visual or hearing impairments; (5) central nervous system diseases (e.g., stroke, Parkinson’s disease). All participants are required to sign a written informed consent form.

### 2.2. Instruments and measurements

A validated demographic survey instrument was administered to document baseline characteristics, encompassing biological parameters (age, sex, BMI-derived metrics), educational background, and additional population-relevant variables. This study employed the Pittsburgh Sleep Quality Index (PSQI), a version validated in Chinese, for assessment. The Cronbach’s α coefficient of this index was 0.84 [19]. This tool quantifies sleep quality via seven psychometrically derived subscales assessing: sleep initiation latency, nightly sleep duration, sleep maintenance efficiency, sleep fragmentation events, self-rated sleep satisfaction, pharmacological sleep aid consumption, and diurnal functional impairment. Component scores (0–3 points each) aggregate to produce a composite index (0–21 points), where higher scores reflect poorer sleep qualitywhere higher scores reflect poorer sleep quality. Globally, a total score of ≤5 is commonly considered indicative of good sleep quality, whereas scores >5 suggest poor sleep quality [20]. The diagnosis of T2D in this study was determined primarily on the basis of standardized medical records from community health centers, and all participants were documented patients with chronic disease management whose diagnostic information was based on the WHO 1999 classification criteria and clearly documented [21].

### 2.3. Sampling method

**Fig 1:**
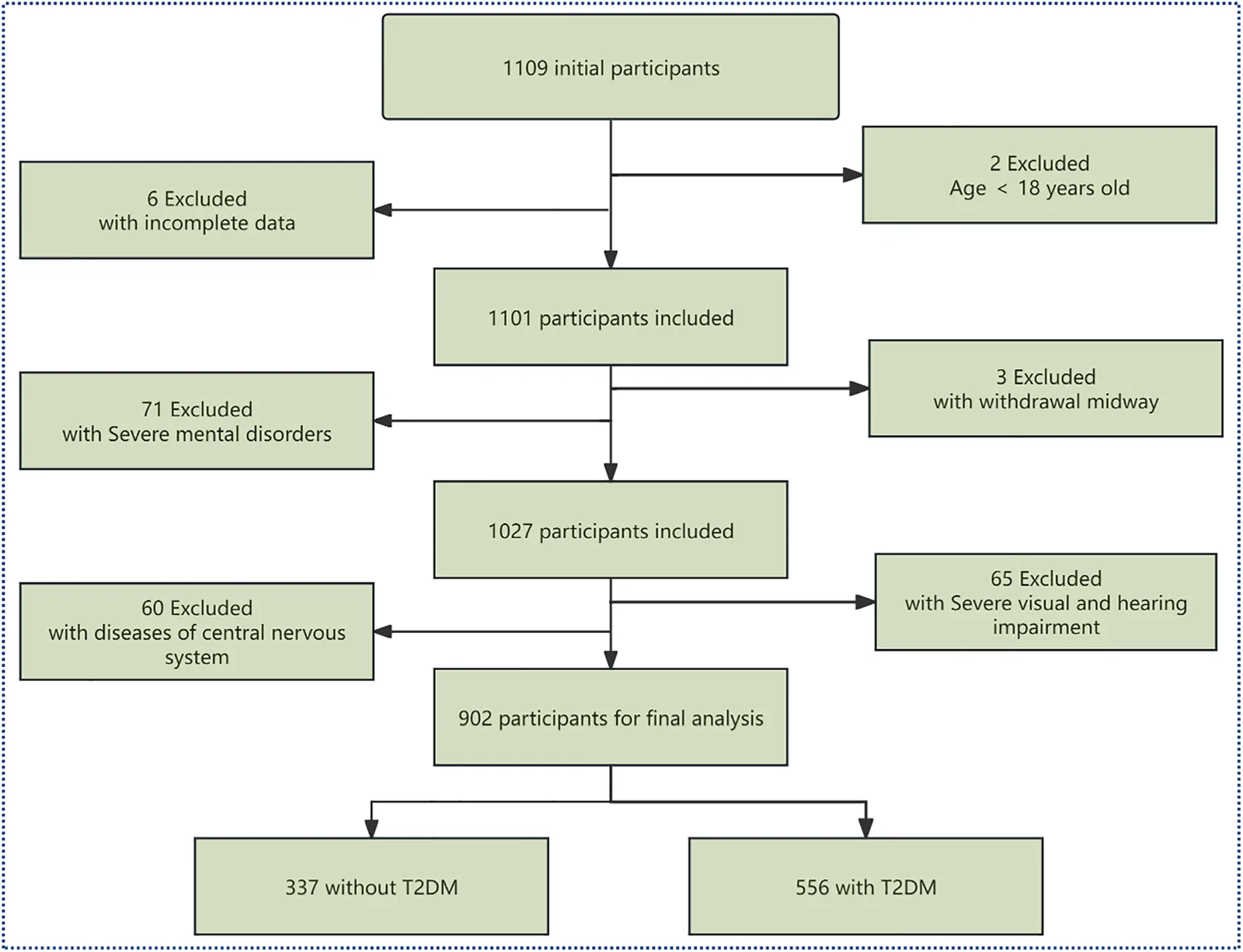
The flow chart of sample collection.

As illustrated in Fig 1, a total of 1,109 participants were initially approached, of whom 902 completed the survey (response rate: 81.3%). Exclusions included age < 18 years (n = 2), incomplete data (n = 6), withdrawal (n = 3), severe mental disorders (n = 71), severe visual/hearing impairments (n = 65), and diseases of central nervous system (n = 60). All investigators received standardized training to ensure consistent data collection. This training regimen ensured uniform administration procedures, including identical wording for questionnaire explanations and standardized response recording methods throughout the study period. The final sample size of 902 exceeded the minimum required sample size of 717, which was calculated using the standard proportion estimation formula (n = Z^2^ × p(1-p)/ e^2^) with the following parameters: p = 0.16 (16% diabetes prevalence based on prior community studies [22]), Z = 1.96 (95% confidence level), and e values ranging from 3% to 10% to capture uncertainty [23]. To enhance robustness, the initial sample size was adjusted upward by 10–20% to account for attrition and incomplete data.

### 2.4. Statistical analysis

The study population was divided into two groups according to whether or not they had been diagnosed with T2D. The normality of all continuous variables (age, BMI, and PSQI score) was assessed using the Shapiro-Wilk test. The results indicated that all continuous variables were normally distributed (Shapiro-Wilk test, all P > 0.05). Continuous variables were expressed as mean ± standard deviation (SD), whilst categorical variables were expressed as percentage. Between-group differences in baseline variables were assessed by means of independent samples t-tests for continuous variables and chi-square tests for categorical variables. Between-group differences in baseline variables were assessed by means of t-tests and chi-square tests. Multiple logistic regression models were developed to express the findings as odds ratio (OR) and 95% confidence interval (CI). Three continuous models were used: model 1 (unadjusted); model 2 (adjusted for age and education level); and model 3 (further adjusted for body mass index, physical activity, smoking status, and alcohol consumption in addition to the covariates in model 2). To analyze the potentially nonlinear association between sleep quality and T2D prevalence, we employed the RCS regression model, a flexible statistical method that allows for the detection of threshold effects and complex dose-response patterns without imposing linearity assumptions [24]. All statistical analyses were performed using R software (version 4.3.3) with a two-tailed p-value threshold of 0.05 for statistical significance.

### 2.5. Ethical approval

The process of obtaining ethical approval and consent to participate is a prerequisite for the initiation of any research study.This study was conducted in accordance with the Helsinki Declaration and received academic ethics review from the College of Health Management of Shanghai Jian Qiao University. The ethics review number is (No: 2024-12-236).

## 3. Results

### 3.1. Basic characteristics of the object of study

Table 1 presents the baseline characteristics of all participants according to the T2D subgroups. In the present study, a total of 902 valid samples were analyzed, of which 565 (62.64%) were found to have T2D. The mean age of the study participants was found to be 59.38 years (±15.09) and 43.02% of them were female. The diabetic group exhibited higher levels of PSQI (11.83 ± 2.02 vs. 9.90 ± 2.72) and BMI (24.51 ± 2.15 vs. 23.96 ± 2.14), and were of a greater mean age in comparison to the non-diabetic group. Furthermore, non-diabetic participants were more likely to have attained higher levels of education, to engage in more frequent exercise, and to currently smoke and consume alcohol. (See Table 1 for details). The schematic representation of specimen acquisition methodology is presented in Fig 1.

**Table 1:** Demographic characteristics and disease-related factors of the study population.

Variables	All participants	Non-T2D	T2D	*P*-value
	n = 902	n = 337	n = 565	
**Gender, n(%)**				0.07
Male	514(56.98)	179(53.12)	335(59.29)	
Female	388(43.02)	158(46.88)	230(40.71)	
**Age, years**	59.38 ± 15.09	50.69 ± 15.75	64.57 ± 12.00	<0.01
**BMI, kg/m²**	24.30 ± 2.17	23.96 ± 2.14	24.51 ± 2.15	<0.01
**Education, n(%)**				<0.01
Illiterate	146(16.18)	18(5.34)	128(22.65)	
Primary school	193(21.39)	51(15.13)	142(25.13)	
Middle school	262(29.05)	82(24.33)	180(31.86)	
High school	145(16.07)	60(17.80)	85(15.04)	
College	119(13.19)	94(27.89)	25(4.42)	
Postgraduate	37(4.10)	32(9.50)	5(0.90)	
**Exercise**				<0.01
Everyday	206(22.84)	126(32.39)	80(14.16)	
At least once a week	176(19.51)	53(15.73)	123(21.77)	
At least once a month	360(39.91)	116(34.42)	244(43.19)	
Sometimes	113(12.53)	31(9.20)	82(14.51)	
Never	47(5.21)	11(3.26)	36(6.37)	
**Smoking**				<0.01
Never smoking	620(68.74)	261(77.45)	359(63.54)	
Current smoking	171(18.96)	44(13.07)	127(22.48)	
Former smoking	111(12.31)	32(9.50)	79(13.98)	
**Drinking**				
Never drinking	637(70.62)	250(74.18)	387(68.50)	<0.01
Current drinking	148(16.41)	35(10.39)	113(20.00)	
Former drinking	117(12.97)	52(15.43)	65(11.50)	
**PSQI score**	11.11 ± 2.49	9.90 ± 2.72	11.83 ± 2.02	<0.01

Notes: Data are presented as mean (SD) or n (%).

### 3.2. Associations between sleep quality and prevalence of T2D

Multiple logistic regression analyses were employed to explore the relationship between PSQI and T2D, and the results are presented in Table 2. A positive association between PSQI (continuous variable) and the occurrence of DM was found in all models, both unadjusted and adjusted. The odds of developing DM increased by 41.1%, 38.3%, and 35.4% for each unit increase in T2D, respectively, from Model 1 to Model 3. Kindly direct your attention to Table 2 for further details.

**Table 2:** Associations between sleep quality and diabetes.

Model	OR	95%*CI*	*P*-value
**Model 1** PSQI score	1.411	(1.322,1.507)	<0.001
**Model 2** PSQI score	1.383	(1.286,1.488)	<0.001
**Model 3** PSQI score	1.354	(1.255,1.461)	<0.001

**Notes:** The initial model (Model 1) did not account for any covariates. Model 2 incorporated adjustments for age and education. Model 3 included comprehensive adjustments for age, BMI, education, exercise, smoking, and drinking.

### 3.3. Restricted cubic spline regression findings

**Fig 2:**
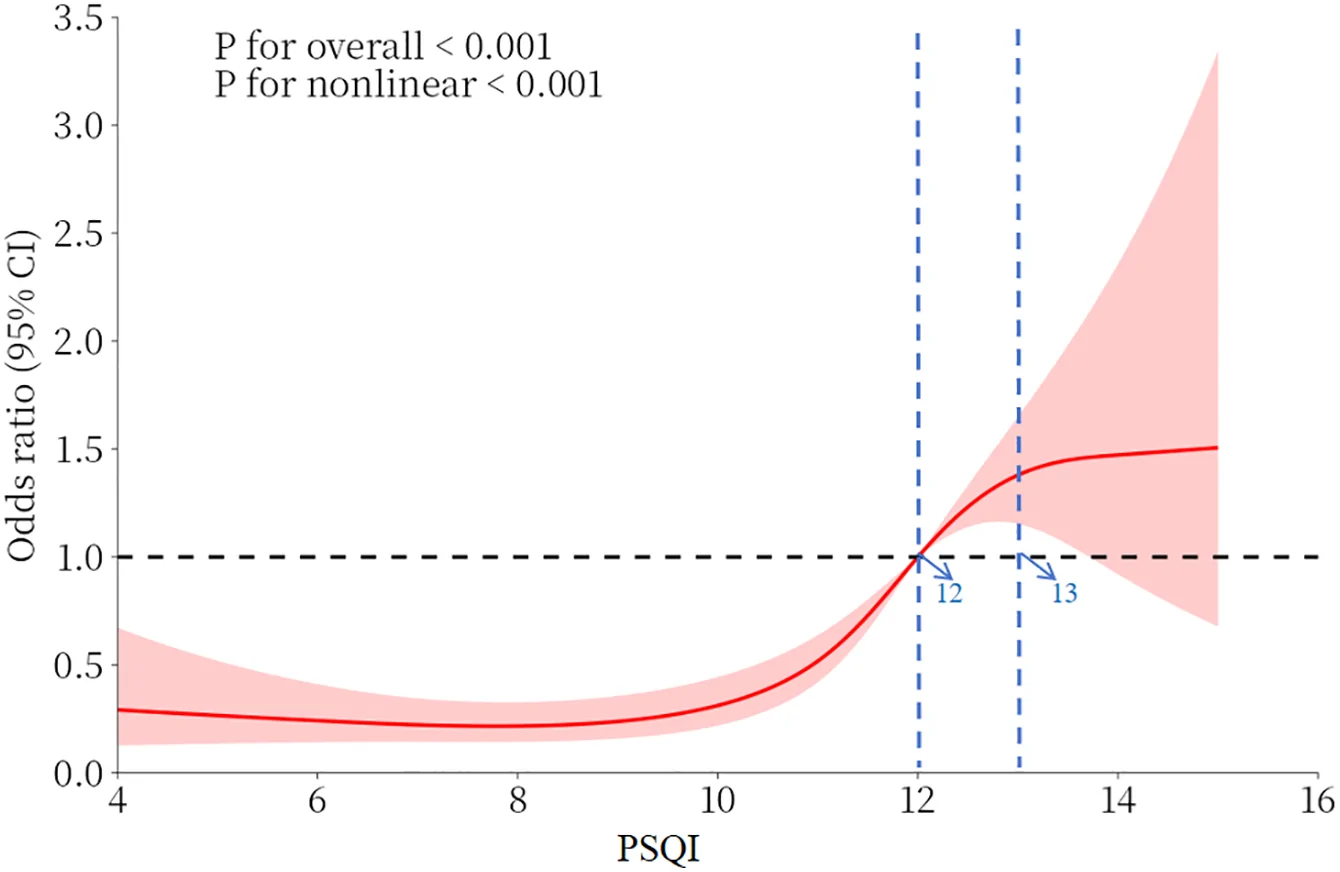
The dose-response relationship between PSQI score and prevalence of T2D.

The covariate-adjusted RCS model revealed a statistically significant nonlinear association between PSQI scores and T2D prevalence (*P*-non linearity <0.001, Fig 2). The spline curve demonstrated two critical threshold values at 12 and 13 PSQI units respectively. The stratified analysis of the population showed that the prevalence of T2D was extremely low among the subjects with a score below the first threshold (PSQI < 12), while those in the middle range (12 ≤ PSQI ≤ 13) exhibited a significantly increased prevalence. These results suggest that maintaining the sleep quality score below 12 might serve as an intervention measure to reduce the prevalence of T2D. To evaluate the robustness of the association between PSQI and the prevalence of T2D, we stratified participants into three distinct categories according to the RCS-derived PSQI thresholds: < 12 (reference), 12–13, and >13. Multiple logistic regression models, with PSQI as a categorical exposure variable, showed consistent cross-sectional associations in both crude and adjusted analyses (Table 3). Participants in both higher PSQI categories (12–13 and >13) had statistically significantly higher odds of prevalent T2D compared with the reference group (all *P* < 0.05; *P* for trend <0.05). These findings support a dose–response pattern between poorer sleep quality and higher odds of prevalence of T2D in this cross-sectional study.

**Table 3:** Relationship between categorized sleep quality measures and prevalence of T2D using RCS-derived thresholds.

Sleep quality categorical	Model 1		Model 2		Model 3	
	OR (95% CI)	*P*-value	OR (95% CI)	*P*-value	OR (95% CI)	*P*-value
<12	1		1		1	
12～13	6.054(4.435,8.263)	<0.001	5.153(3.648,7.278)	<0.001	4.644(3.259,6.619)	<0.001
>13	5.848(3.461,9.883)	<0.001	5.368(2.920,9.870)	<0.001	4.514(2.426,8.399)	<0.001
P for trend	<0.001		<0.001		<0.001	

**Notes:** The continuous PSQI measure was discretized into three clinically meaningful categories using threshold values derived from RCS modeling.

## 4. Discussions

In this study, the PSQI total score was used as a composite measure of sleep quality. The findings of the present study demonstrated a positive association between PSQI scores and the OR of prevalence of T2D. Furthermore, it was observed that patients diagnosed with T2D exhibited significantly higher sleep quality scores in comparison to those without T2D. Higher PSQI scores were found to be associated with greater odds of T2D, even after adjusting for relevant confounding factors.This is consistent with the research results of Li [25] and others. Poor sleep quality may elevate T2D risk through dual physiological pathways: primarily by diminishing insulin sensitivity and, secondarily, impairing glucose tolerance. These combined metabolic disturbances result in sustained hyperglycemia, consequently increasing susceptibility to T2D [26,27].

Lee [28] and others assessed sleep quality using the PSQI and analyzed its association with T2D. However, in their study, PSQI scores were categorized into two groups (impaired sleep group vs. non-impaired sleep group). In contrast, our study treated PSQI as a continuous variable and employed a RCS model. The analysis revealed the existence of a threshold range for the PSQI scores, which was further confirmed by subgroup analyses. Specifically, when PSQI scores exceeded 12, the OR progressively increased with higher PSQI scores, indicating an increased prevalence of T2D. Notably, the OR plateaued at a PSQI score of 13, suggesting that the rate of increase in T2D prevalence slows after surpassing this threshold.

However, the interpretation of this plateau warrants specific caution. A critical consideration is the well-documented, elevated prevalence of obstructive sleep apnea (OSA) among individuals with T2D [29]. OSA itself is a potent disruptor of sleep architecture and continuity (leading to higher PSQI scores) and a known independent risk factor for insulin resistance and the development/ progression of T2D [30,31]. Therefore, we cannot exclude the possibility that the observed plateau may partly reflect the growing contribution of underlying, unmeasured OSA (or other specific sleep disorders) within the diabetic cohort, particularly at higher PSQI levels where OSA is more likely to co-occur. While the plateau may also represent a genuine biological threshold effect, the potential confounding influence of OSA, intrinsically linked to the diabetic state, remains a key methodological challenge in this analysis and an important avenue for future research.

Sleep constitutes a modifiable risk factor in disease pathogenesis. The study findings suggest that sleep quality parameters should be systematically incorporated into T2D prevention strategies for comorbid patients. However, the potential mediating role of conditions like OSA underscores the importance of refined assessment; interventions targeting sleep quality for diabetes risk reduction may need to specifically address OSA where present. Implementation of sleep hygiene protocols aimed at reducing sleep latency, optimizing sleep maintenance efficiency, and mitigating diurnal hypersomnolence remains a foundational approach and should be prioritized as a key preventive measure against T2D progression in populations with multimorbidity. Previous studies have mostly used categorical variables to discuss the relationship between sleep quality and T2D, or explored the linear relationship between PSQI scores and T2D, which limits the full explanation of the relationship between PSQI and T2D [7,32,33]. In contrast, the RCS model is a nonlinear regression model that captures the nonlinear relationship between the independent and dependent variables more flexibly and has strong explanatory power [17,34]. T2D is a common endocrine and metabolic disease that can lead to a variety of complications, causing great suffering and economic burden to patients [4,5]. Early recognition and effective intervention are essential for the prevention of T2D.

This study find that a PSQI score greater than 12 is associated with higher prevalence of T2D. While these findings are preliminary, they suggest a potential association that warrants further investigation. Crucially, future research must incorporate objective sleep measures capable of detecting OSA to disentangle the specific role of sleep quality per se from coexisting sleep disorders such as OSA. Similarly, future research should explore whether comprehensive sleep quality assessment including OSA screening could be integrated into T2D screening protocols, pending external validation of these thresholds. We caution that endocrinology clinics should await confirmatory and more comprehensive evidence, ideally incorporating objective sleep monitoring, before equipping standardized PSQI questionnaires. Should subsequent studies replicate our findings, targeted assessment of OSA followed by appropriate treatment, alongside sleep hygiene interventions, might be considered to intervene in high-risk populations before impaired glucose tolerance occurs.

## 5. Strengths and limitations

This study adopted a cross-sectional design, which can effectively describe the correlation characteristics between sleep quality and T2D. However, it cannot establish a temporal sequence, thereby limiting the inference of causal relationships. A critical challenge is the potential for reverse causality: T2D and its complications may contribute to sleep disturbances (e.g., nocturia, neuropathic pain), rather than poor sleep quality solely driving T2D development.

Critically, a major limitation arises from group differences in key characteristics (Table 1). The diabetic cohort was significantly older, had higher BMI, lower education, reduced exercise, and increased smoking/drinking. Collectively, these factors are established predictors of poor sleep (e.g., age-related sleep fragmentation, nicotine/alcohol disruption) and acted as confounders. Although statistically adjusted, their marked imbalance suggests possible residual confounding.

Alternatively, unmeasured common causes may underlie both conditions, such as disease duration, glycemic control indices, lifestyle, social support, medication use, depression, sleep apnea, and comorbidity severity,etc. Future research should conduct prospective cohort studies, which are crucial for further clarifying the potential causal relationship between sleep quality and T2D. The sample size of this study was 902 cases, and it only included data from Shanghai and Jiangsu. It may not fully represent the actual situation of all patients with chronic diseases. Future research should expand the sample size through multi-center, stratified random sampling and include more regions, medical institutions, and disease spectra to improve the reliability of the results.

## 6. Conclusion

These findings suggest an association between poor sleep quality and the prevalence of T2D; however, prospective studies are required to establish temporal sequence and causality. While a critical range appears to exist for this association, this threshold is exploratory and derived from a single cross-sectional study without external validation. Instead, sleep quality assessment using PSQI thresholds requires validation before clinical application, but may theoretically serve as a marker for identifying high-risk individuals who warrant closer monitoring for T2D and its complications. Moreover, patients with chronic diseases should be encouraged to evaluate their sleep quality as part of comprehensive chronic disease management.

## Supporting information

S1 File Data. (XLSX)

## Abbreviations

T2D: type 2 diabetes
COPD: Chronic Obstructive Pulmonary Disease
RCS: Restricted Cubic Spline
PSQI: Pittsburgh Sleep Quality Scale
BMI: Body Mass Index
SE: standard error
OR: Odds Ratio
CI: Confidence Intervals
OSA: Obstructive Sleep Apnea
